# Providing a Web-based Online Medical Record with Electronic Communication Capabilities to Patients With Congestive Heart Failure: Randomized Trial

**DOI:** 10.2196/jmir.6.2.e12

**Published:** 2004-05-14

**Authors:** Stephen E Ross, Laurie A Moore, Mark A Earnest, Loretta Wittevrongel, Chen-Tan Lin

**Affiliations:** ^1^Division of General Internal MedicineUniversity of Colorado Health Sciences CenterAurora COUSA; ^2^Colorado Health Outcomes ProgramUniversity of Colorado Health Sciences CenterAurora COUSA

**Keywords:** Heart Failure, Congestive, Patient Participation, Patient Advocacy, Patient Compliance, Internet, Randomized Controlled Trial, Electronic Communication, Electronic Health Record

## Abstract

**Background:**

It is possible to provide patients with secure access to their medical records using the Internet. Such access may assist patients in the self-management of chronic diseases such as heart failure.

**Objective:**

To assess how a patient-accessible online medical record affects patient care and clinic operations. The SPPARO (System Providing Access to Records Online) software consisted of a web-based electronic medical record, an educational guide, and a messaging system enabling electronic communication between the patient and staff.

**Methods:**

A randomized controlled trial was conducted in a specialty practice for patients with heart failure. Surveys assessing doctor-patient communication, adherence, and health status were conducted at baseline, 6 months, and 1 year. Use of the system, message volume, utilization of clinical services, and mortality were monitored.

**Results:**

One hundred and seven patients were enrolled (54 intervention and 53 controls). At 12 months, the intervention group was not found to be superior in self-efficacy (KCCQ self-efficacy score 91 vs. 85, p=0.08), but was superior in general adherence (MOS compliance score 85 vs. 78, p=0.01). A trend was observed for better satisfaction with doctor-patient communication. The intervention group had more emergency department visits (20 vs. 8, p=0.03), but these visits were not temporally related to use of the online medical record. There were no adverse effects from use of the system.

**Conclusions:**

Providing patients with congestive heart failure access to an online medical record was feasible and improved adherence. An effect on health status could not be demonstrated in this pilot study.

## Introduction

Even before electronic medical records became available, there was interest in encouraging patients to review their medical records [1]. In doing so, researchers sought to educate, engage, and empower patients. At the same time, researchers recognized that the medical record contains technical language and raw data that was never intended for the layman, so the medical record might also worry or confuse patients [2]. Clinical trials that gave medical patients access to their written records showed modest benefits (such as improved doctor-patient communication) with minimal risk of harm [3- 6]. These studies were limited, however, by small sample sizes, lack of randomized controls, short duration of exposure to the medical record, and use of non-standardized instruments for assessment of outcomes.

With the advent of electronically stored medical records and of the Internet, it has become technically feasible to provide patients access to their records online. In comparison to a written medical record kept in centralized storage, an Internet-accessible medical record may be particularly helpful for patients. Patients can review an online medical record repeatedly at their convenience, in the context of other resources that may assist them in comprehending it. Demonstration projects have shown that patients can be provided access to online medical records without compromising privacy and security. Furthermore, access to these records is appreciated by patients and causes little disruption to clinical operations [7- 9]. Controlled trials of this intervention, however, have not yet been reported.

The aim of our study was to assess the effects of a patient-accessible online medical record in a rigorously controlled fashion. Our version, System Providing Patients Access to Records Online (SPPARO), provides access to clinical notes and test results, and also provides a method of sending electronic messages to the clinic staff. We sought to determine whether access to SPPARO would improve patient satisfaction, adherence, and health status. We also studied whether providing access to SPPARO would affect the clinical workload. In addition, to assess the reach of the intervention, we obtained information from the patients who were offered SPPARO, but declined to use it.

We chose to intervene in a specialty clinic for heart failure in order to study a set of patients who shared a common medical condition and were likely to benefit from reading their medical records. Because patients with heart failure often require frequent visits and complicated medical regimens, we anticipated that access to medical records would be particularly helpful for these patients, by clarifying their doctors' assessments and instructions. We hypothesized that access to the medical record would improve their self-efficacy, adherence, and satisfaction, and might improve their health status as well.

## Methods

### Setting

We conducted the clinical trial in a specialty clinic for patients with heart failure at University of Colorado Hospital in Denver, Colorado. The majority of patients in the practice have New York Heart Association Class II or Class III symptoms of heart failure. Patients in this practice are cared for by a team of physicians. They therefore receive clinical notes from a variety of physicians over the course of their treatment.

The Colorado Multiple Institutional Review Board approved the study design. Security systems, including the use of passwords, firewalls, and encryption were used to prevent unauthorized access to the medical record. All participating patients signed an informed consent that included information on how to protect the privacy of the medical record. All physicians in the practice gave informed consent for their clinical notes to be shared during the study period, as well as reports of laboratory, radiology, and procedure results.

### Recruitment of Study Participants

Patients were eligible for the study if they were followed in the practice, spoke English, and were 18 years of age or older. They needed to have used a Web browser before, although they did not need to have access to the Internet at home. Physicians, nurses, physician assistants, and nurse practitioners were excluded, since their sophistication in interpreting information from the medical record would not reflect the typical user of the system.

In August 2001, a recruitment letter explaining the study was sent to eligible patients. From September 2001 through December 2001, our research assistant approached patients in the waiting room of the practice, asking them if they would be interested in reading their medical records online in the context of a study that would provide this by random assignment.

After enrollment of participants in the primary study was completed, we then surveyed the patients who had declined to participate ("decliners"). After an initial solicitation by mail, patients who had not enrolled were solicited to complete written questionnaires in the clinic's waiting room from April 2002 through September 2002.

### Randomization

After completing the informed consent, patients who were interested in enrolling in the primary study were provided with an enrollment form and the initial questionnaire. When patients completed the initial questionnaire they were blinded to their enrollment status.

As the questionnaires were received, patients were consecutively assigned identification numbers that were linked to either the intervention group or the control group according to a predefined computer-generated randomization scheme developed by a statistical consultant. Randomization was restricted so that equal numbers of patients were assigned to the intervention and the control groups in blocks of 10.

### Intervention

Participants in the intervention group were given a user identification and password to SPPARO (System Providing Access to Records Online). These participants also received a written user guide to the system. Patients in the control group continued to receive standard care in the practice. They were offered use of SPPARO after the study was completed as an incentive to participate.

SPPARO provides a secure Web interface to three components: the medical record, an educational guide, and a messaging system (see Multimedia Appendix). Security was provided through Secure Socket Layer 128 bit encryption for all transactions beginning with login. SPPARO retrieves data from the hospital's clinical data repository (3M Lifetime Data Repository, St. Paul, MN), which is kept behind a firewall. The medical record consists of clinical notes, laboratory reports, and test results (including reports of radiographs and echocardiograms). The clinical notes were dictated by physicians and transcribed after every office visit. All clinical notes from the physicians in the heart failure practice from the start of the study period onward were available. The educational guide is an online version of the printed materials that all patients in the heart failure practice receive at their first visit. The messaging system allowed patients to exchange secure messages with the nursing staff in the practice.

The physicians and practice staff were not told which patients were enrolled into the study. They could become aware of a patient's enrollment status, however, if a patient directly mentioned using it, or if a patient sent an electronic message using SPPARO.

During the study, periodic messages were sent by the research staff to all participants. Participants were informed about upcoming surveys, and were encouraged to contact the research assistant if they had a change of address of telephone number. In addition, patients in the intervention group were reminded to call the research assistant if they had problems using SPPARO.

### Data Collection

#### Use of SPPARO/Electronic Messaging

Throughout the study period we tracked the dates that participants used SPPARO and what components were used. The unit of analysis was a "patient hit day," which was defined as a day that a particular participant used a component of SPPARO. Thus, if a single participant used a component of SPPARO multiple times on a given day, this counted as a single "patient hit day" for that component.

We tracked messages sent to the practice through the SPPARO system and categorized them based on content. We also tracked phone messages from participants through review of the written medical record and through logs kept by the nursing staff.

#### Questionnaires

For the primary study, participants completed written questionnaires at baseline, 6 months, and 12 months. The 6-month and 12-month questionnaires were mailed.

The baseline questionnaire assessed sociodemographic characteristics. All questionnaires included assessments of health status, patient satisfaction, and self-reported compliance. We used previously validated survey instruments when available. Health status was assessed using the Kansas City Cardiomyopathy Questionnaire (KCCQ) [10]. Patient satisfaction with doctor-patient communication was assessed using the Art of Medicine Questionnaire (HealthCare Research, Inc., Denver, CO, USA) [11]. Questions were modified to reflect the care provided by the panel of doctors, rather than the care of a specific doctor or a specific clinical encounter. A 5-point categorical response scale was used in place of a 9-point semantic differential scale. Adherence to medications was assessed using the questions derived from Morisky [12], and general adherence to medical regimens was assessed from the General Adherence Scale from the Medical Outcomes Study (MOS) [13].

The written questionnaire for the "decliners" assessed sociodemographic characteristics. Health status was assessed using a modification of the KCCQ symptom score. Most of these questionnaires were completed in the clinic's waiting room.

#### Mortality and Utilization of Health Services

Information on mortality came from chart review, the nursing staff, and telephone and mail contact with the homes of patients who had not returned follow-up questionnaires. Information on emergency department visits and hospitalizations at the University of Colorado Hospital came from chart review. The nursing staff from the practice also kept a weekly log of the time they spent answering messages sent through the SPPARO messaging system.

### Outcome Measures and Sample Size

The primary endpoint of the study was a change in the self-efficacy domain of the Kansas City Cardiomyopathy Questionnaire. Like the other domains of the KCCQ, this domain generated a scaled score from 0-100. We chose a change of 7.7 to be the minimal clinically significant difference in this measure, based on a validation study of the KCCQ, which found that the mean difference in self-efficacy score during and 3 months after hospitalization for congestive heart failure was 15.4 points [10]. We set our criterion of clinical significance to be half this difference. Based on the validation study's standard deviation of change of 18.5 , we derived a target enrollment of 100 patients per group, which would provide 80% power to detect a difference of 7.7 points on the KCCQ self-efficacy domain at the p<0.05 significance level, using a two-sided test.

We did not set *a priori* thresholds of clinical significance for the other outcome measures. However, a change of 5 points on KCCQ scale scores, either as a group mean or as an intra-individual change, is considered clinically important (Rumsfeld J, Masoudi F, personal communication). For patient satisfaction, a difference of 0.25 points in the mean 9-point summary score from the Art Of Medicine survey, equivalent to a change of 0.14 points in our 5-point Likert scale, was previously considered to be a minimally significant difference [11]. For adherence, although the Morisky score and the MOS General Adherence score have been shown to be valid measures of adherence [12] and have been associated with clinical measures of disease activity and control [14,15], no minimal threshold of clinical significance has been established.

### Statistical Analysis

Baseline comparisons were made between the intervention and control groups and between participants and decliners using t-tests and Chi-square tests. For insurance status, patients were considered to be in a "safety net" program if they had no insurance, were enrolled in a state assistance program for needy patients, or were enrolled in Medicaid.

Utilization of health services (number of hospitalizations, emergency department visits, clinic visits, and messages sent to the clinic) was analyzed in several ways because of the skewed nature of the data:

1. The proportion of patients who utilized a service was compared using Chi-square and Fisher's exact test.
2. The mean number of utilizations per patient was compared using the Wilcoxon Rank-Sum test. The number of messages sent per patient was also transformed using square root, and means were compared using t-test.
3. Mean monthly message volume was compared using paired t-tests.

For scored questionnaire items, we used a repeated measures analysis for incomplete data to test whether the groups diverged from baseline to the 6-month and 12-month questionnaires. (A mixed model analysis was performed using PROC MIXED from the SAS statistical package, version 8.1, SAS Institute, Cary, NC, USA). The repeated measures model for incomplete data used observations prior to dropout to adjust the 6-month and 12-month means for each outcome measure, under the assumption that data were missing at random. The adjustment made to the 6-month and 12-month outcome measures was based on (1) the previously observed values of the outcome measure in the censored subjects, and (2) the strength of the association between previously observed values and the 6-month and 12-month measures in the uncensored subjects. This implicitly assumed that the associations observed among the baseline, 6-month, and 12-month measures in the uncensored subjects would have been observed in the censored subjects [16].

## Results

### Enrollment, Retention, and Demographics

Out of 394 patients in the practice panel, we enrolled a total of 107 participants (27%), 54 in the intervention group and 53 in the control group. We capped enrollment from the heart failure practice in December 2001, when we reached a point of maximal recruitment from the waiting room of the practice. Unfortunately, this point was reached before we were able to achieve our target enrollment.

**Figure 1:**
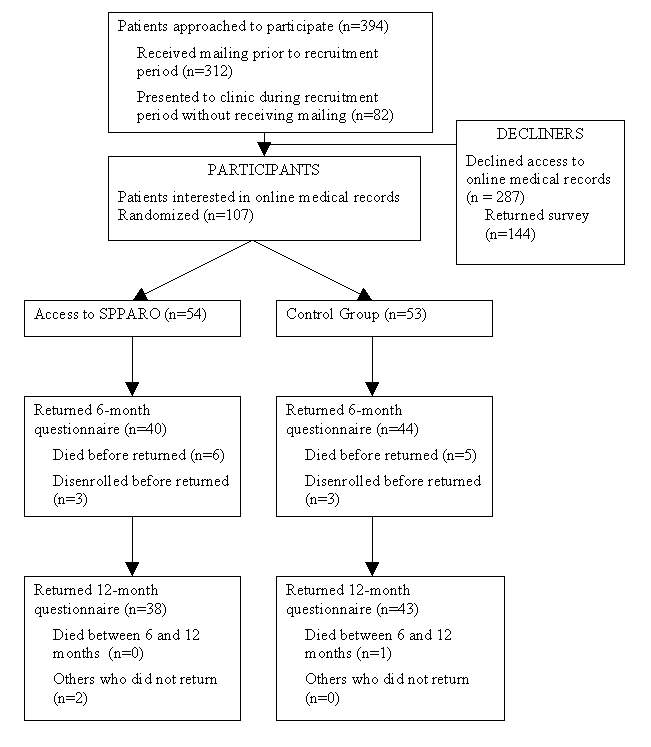
Flow of Participants Through The Study

Figure 1 illustrates the flow of participants through the study. The pool of eligible patients was derived from the practice census at the beginning of the study and subsequent records of patients who had appointments during the enrollment period. Two interested patients were excluded because they were health professionals (one physician's assistant and one nurse practitioner). Approximately 10 patients were not approached for enrollment because they did not speak English. Of the patients enrolled, 78.5% remained at 6 months and 76% remained at 12 months.

After recruitment was completed, we identified a pool of 288 patients who were cared for by the practice during the recruitment period but did not enroll in the primary study. Of these, 144 (50%) completed the "decliners" survey.

Table 1 summarizes the characteristics of the intervention, control, and decliner groups. At baseline, the intervention and control groups did not differ in their socioeconomic characteristics, or in their health status as assessed by the KCCQ symptom score. Although we did not use the New York Heart Association (NYHA) heart failure classification, our "participants" (the combination of the intervention and the control groups) had a mean KCCQ symptom score of 65, similar to the mean score for patients with NYHA Class II symptoms in the KCCQ validation study [10].

**Table 1:** Baseline Demographic Characteristics *

Variable	Participants (n = 107)		Decliners (n=144)	Participants vs. Decliners p-value
	Intervention Group (n = 54)	Control Group (n = 53)		
Mean age (years)	57	55	58	0.12
Male	80%	74%	64%	0.19
Self-Efficacy (from KCCQ)†	86	83	83	0.56
Symptom Score (modified KCCQ)†	69	60	61	0.15
College graduate	53%	44%	26%	< 0.001
White, non-Hispanic	92%	88%	75%	< 0.01
Household income < $45,000/year	56%	50%	76%	< 0.001
Safety-net insurance program	19%	19%	37%	< 0.01
Previous experience using the Internet	100%	100%	48%	< 0.001
Access to home computer	96%	94%	56%	< 0.001

^*^ Participants and decliners are compared using t-test for continuous variables, and chi-squared for dichotomous variables.

^†^ KCCQ = Kansas City Cardiomyopathy Questionnaire

**Figure 2:**
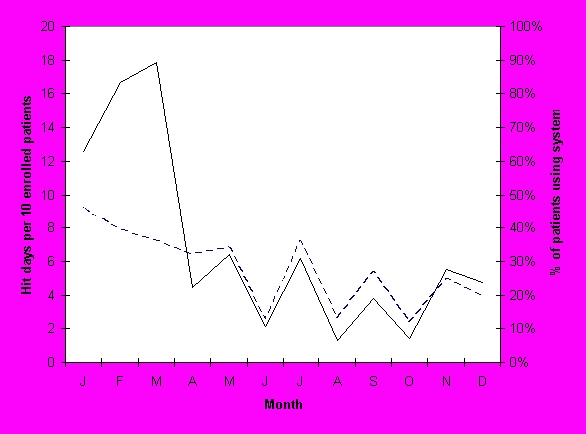
Monthly Use of SPARRO Over Study Period

Decliners did not differ from participants in their age and gender distribution, their health status, or their self-efficacy. Compared with participants, however, decliners had lower incomes, and a lower percentage were white or of non-Hispanic race/ethnicity. Furthermore, fewer had standard medical insurance, or a college education. Although they were less likely than participants to have experience with the Internet, roughly half of the decliners nonetheless did have access to a computer and experience with the Internet.

### Use of SPPARO/Electronic Messaging

The number of patients using SPPARO and the number of patient hit days are presented in Figure 2. Use of SPPARO was highest in the first 3 months after enrollment, then leveled off. After the first 3 months, an average of 24% of the enrolled patients used SPPARO in a given month. During this time interval, frequency of use of SPPARO averaged 0.4 hit-days per enrolled patient per month. This was approximately 1 hit-day per clinic visit.

Solid line indicates hit days per 10 intervention patients per month. Dashed line indicates the percentage of the intervention patients that used SPPARO (System Providing Patients Access to Records Online) each month. Monthly website activity is normalized to account for attrition over the course of the study.

**Figure 3:**
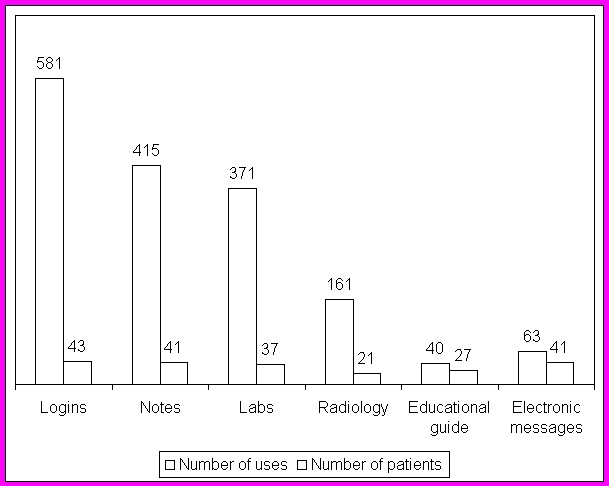
Cumulative Use of SPARRO Over the 12-Month Study Period

**Figure 4:**
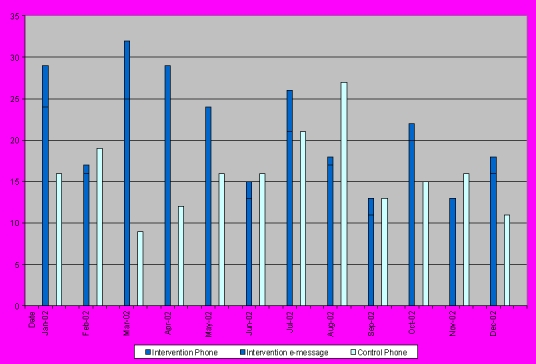
Monthly Message Volume

Cumulative use of SPPARO over the study period is shown in Figure 3. Subjects most commonly reviewed clinical notes and laboratory results, and did so repeatedly. Fewer subjects reviewed radiology results, but those who did also reviewed them repeatedly. The educational guide was reviewed least frequently, and was generally reviewed only once.

The electronic messaging function in SPPARO appeared to supplement, rather than replace, telephone messages. The intervention group sent more messages to the practice (350 total: 287 phone calls and 63 electronic messages) than the control group (267 phone calls) over the course of the study. The number of total messages (phone + electronic messages in the intervention group, phone messages in the control group) sent per month are compared graphically in Figure 4. The number of total messages sent per month did not show a statistically significant difference (p=0.70). The number of messages sent per patient did demonstrate a statistically significant difference when analyzed by square root transformation (p=0.02). The difference was more pronounced during the first 6 months of the intervention (150 messages in the intervention group vs. 88 in the control group, p=0.05) than the second 6 months (109 messages vs. 103, p=0.66). The main categories of messages overall were to schedule appointments (20% of total messages), to refill medications (15%), to ask questions about medications (14%), to get test results (12%), to report feeling ill (8%), and to get assistance interpreting test results (3%). In none of the individual categories was there a statistically significant difference in call volume between the intervention and the control group.

Nurses spent a total of 304 minutes answering computer messages over the course of the 12 months, a mean of 5.6 minutes per subject per year. In interviews, the physicians and nursing staff did not feel that providing SPPARO to the intervention group resulted in a perceptible change in their workload.

### Self-Efficacy, Health Status, Adherence, and Patient Satisfaction

Repeated measures of self-efficacy, health status, adherence, and patient satisfaction are presented in Table 2. For our primary outcome, the self-efficacy domain of the KCCQ, there was a trend towards an improvement in the intervention group, but the improvement of 6 points did not reach the threshold value of 7.7 that we had set as a standard for this outcome. (Based on actual enrollment, the study had a power of 73% to detect a difference of 7.7, and 80% power to detect a difference of 8.8, with a two-sided alpha of 0.05). For the other measures of health status from the KCCQ, there were trends towards improvement in a many domains, but no statistically significant improvements were demonstrated when adjusted for multiple comparisons.

General adherence to medical advice showed significant improvement in the intervention group compared with the control group. Adherence to medications showed a similar trend but did not reach statistical significance.

Patient satisfaction with doctor patient-communication demonstrated a trend towards improvement in two areas: how well patients felt their problems were understood, and how well doctors explained information. While significant results were found for these two items individually, the findings did not reach statistical significance when adjusted for multiple comparisons. There was no significant improvement in the other patient satisfaction domains.

### Mortality and Utilization of Health Services

Table 3 compares mortality, hospitalizations, Emergency Department visits, and practice visits for the intervention and control groups. Although the number of patients who visited the emergency department did not differ significantly, there was a significant increase in the number of overall emergency department visits in the intervention group (20 visits) relative to the controls (eight visits). Of the emergency department visits in the intervention group, only four occurred within 7 days of using SPPARO.

Proportions of patients in the two groups are compared using Chi-squared and Fishers' Exact Test. The number of utilizations in the two groups is compared by comparing the number of utilizations per patient using the Wilcoxon rank-sum test.

**Table 2:** Changes In Adherence, Health Status, And Patient Satisfaction Over Time

Measure		Baseline	6 months		12 months		p-value
			Intervention - Control	Difference (CI)	Intervention - Control	Difference (CI)	
**Health Status (KCCQ Domains), scored from 0 to 100**							
	Self-efficacy	85	88 - 84	+4 (-3, 9)	91 - 85	+ 6 (-1, 11)	0.08
	Symptom stability	49	45 - 49	-4 (-15, 6)	63 - 46	+17 (4, 29)	<0.01†
	Symptoms	63	61 - 65	-4 (-11, 3)	64 - 65	0 (-8, 8)	0.96
	Quality of life	56	64 - 59	+5 (-5, 13)	64 - 62	+2 (-7, 11)	0.63
	Functional status	66	63 - 69	-6 (-12, 0)	67 - 70	-3 (-11, 3)	0.31
	Clinical summary	64	62 - 66	-4 (-10, 2)	69 - 66	-3 (-10, 4)	0.38
	Physical limitations	66	63 - 70	-7 (-13, -1)	69 - 73	-4 (-12, 3)	0.26
**Adherence**							
	Medication Adherence (scored from 0 to 4)	3.4	3.5 - 3.4	+0.1 (-0.2, 0.4)	3.6 - 3.4	+0.2 (-0.1, 0.6)	0.15
	General Adherence (scored from 0 to 100)	82	81 - 78	+2.3 (-3.7, 8.3)	85 - 78	+6.4 (1.8, 10.9)	0.01*
**Patient Satisfaction, scored from 1 to 5**							
	Overall, how well do the heart doctors understand your problems?	4.5	4.4 - 4.4	0 (-0.3, 0.2)	4.6 - 4.2	+0.4 (0.1, 0.6)	0.02§
	Overall, how well do the heart doctors explain to you what they are doing and why?	4.2	4.5 - 4.1	+0.4 (0.1, 0.7)	4.5 - 4.1	+0.4 (0.1, 0.7)	0.02§
	Overall, how well do the heart doctors speak to you using words that are easy for you to understand?	4.2	4.2 - 4.3	-0.1 (-0.4, 0.1)	4.1 - 4.3	-0.2 (-0.5, 0.1)	0.15
	Overall, how well do the heart doctors listen to your concerns and questions?	4.5	4.6 - 4.3	+0.3 (0.02, 0.5)	4.5 - 4.3	+0.2 (-0.1, 0.5)	0.26
	Overall, how much confidence do you have in the ability or competence of the heart doctors?	4.5	4.6 - 4.4	+0.2 (-0.1, 0.4)	4.5 - 4.5	0 (-0.2, 0.3)	0.80
	Overall, how satisfied are you with the service that you received from the heart doctors?	4.5	4.5 - 4.5	0 (-0.2, 0.3)	4.6 - 4.4	+0.2 (-0.2, 0.5)	0.07‡

^*^ p = 0.02 when adjusted for multiple comparisons

^†^ p = 0.06 when adjusted for multiple comparisons

^§^ p = 0.13 when adjusted for multiple comparisons

^‡^ p = 0.30 when adjusted for multiple comparisons

The changes in outcome measures in the intervention group at each time interval are compared to the corresponding changes in the control group. Statistical analysis uses a repeated measures approach, with a mixed model to account for censored patients.

**Table 3:** Mortality, Hospitalizations, Emergency Department Visits, and Clinic Visits During Study Year 2002

		Intervention Group	Control Group	p-value
	Deaths	6 (11%)	6 (11%)	1.00
**Hospitalizations**				
	Number of patients	11 (20%)	12 (23%)	0.81
	Number of hospitalizations	22	21	1.00
**Emergency Room**				
	Number of patients	11 (20%)	7 (13%)	0.44
	Number of visits	20	8	0.03
**Heart Failure Practice**				
	Number of patients	50 (93%)	49 (92%)	1.00
	Number of visits	324	325	0.66

### Adverse Effects

There were no reports of adverse effects resulting from use of SPPARO. In only one case did access to SPPARO result in a patient complaint. That patient did not agree with a statement regarding his alcohol consumption. He requested that an amendment be placed in the clinical notes, and his concerns were documented.

## Discussion

### Principal Findings

Overall, this randomized controlled trial demonstrated that an Internet-accessible medical record can be provided to chronically ill patients without disrupting clinical practice, and may offer modest benefits. Although we did not demonstrate a significant effect on our primary outcome, self-efficacy, there was an improvement in general adherence to medical advice, and there were trends towards improvement in patient satisfaction with doctor-patient communication. Both adherence and doctor-patient communication are important issues in the management of complex chronic diseases such as heart failure. Although we did not demonstrate improvements in overall health status in this study, the study was not powered to exclude the possibility of such improvements.

Two other statistically significant findings are of dubious clinical significance. Although the intervention group demonstrated a dramatic improvement in symptom stability between 6 and 12 months, it seems implausible that this measure, which is based on a single item in the survey, represents an important clinical outcome when the other KCCQ domains remained unchanged. Likewise, although emergency department utilization was significantly higher in the intervention group, the intervention group did not differ from the control group in hospitalizations or mortality. It seems implausible that use of SPPARO would be the cause of increased emergency department visits without a temporal relationship between the events, or a more consistent increase in use of health services overall.

Including the electronic messaging system did result in a significant increase in the number of messages sent to the practice, particularly in the first 6 months of use. Neither the nurses nor the physicians perceived an increase in workload.

### Comparisons to Other Studies

Our results were generally consistent with previous studies of patient-accessible medical records [1]. Several of these studies have also demonstrated improvements in adherence [17,18] and satisfaction with doctor-patient communication [19,20] . Most studies also did not find that patient-accessible medical records increased subjective workload [4-6,19], although a randomized trial of hospitalized patients demonstrated increased time spent answering patient questions [3]. With regard to the use of SPPARO over time, we found that use of the system was initially high, and then leveled off. This pattern has been seen in other patient-centered information technology programs, such as D-Net [21]. To some degree, this may have represented greater efficiency in use of the system, as patients learned that information was not updated unless they had a clinical encounter or laboratory study, and as they learned how long it took for transcribed notes and laboratory reports to appear. There may also have been an initial novelty effect that waned over time. With regard to electronic messaging, we found that electronic messaging did not substitute for phone communication, which is similar to the study by Katz [22].

### Strengths and Weaknesses

In comparison to previous studies, this study is notable for its rigorous design. By obtaining a participant pool of only those who were interested in online patient-accessible medical records, and randomly assigning exposure status within that pool, we were able to minimize selection bias and maximize internal validity. Nonrandomized studies of interventions using information technology may be particularly prone to selection bias. In our own study, there were clear socioeconomic differences between the participants and the decliners, although their age, gender composition, and health status were not significantly different. Selecting patients with a homogeneous, serious disease process also facilitated the study of outcome measures such as mortality, symptoms, and quality of life (albeit at some cost to the study's generalizability.) The primary weakness of the study was its small sample size, which limited its power to detect effects of the intervention, particularly after the attrition in the first 6 months.

### Implications

Overall, this trial suggests that a patient-accessible electronic medical record can be implemented with the potential for a modest benefit in adherence and minimal impact on clinic operations. Although the majority of patients were not interested in online medical records, the fact that fully one-quarter of the patients in the practice were interested demonstrates that this intervention can appeal to a substantial number of patients, demonstrating its "reach" [23]. However, results may vary in more heterogeneous practices (such as primary care practices). Patients with more acute illnesses, or less severe chronic illnesses, may only access an online medical record sporadically, so the effects of the intervention may be less robust. Providing access to clinical notes that address mental health issues may also be more problematic [24- 26] .

The overall impression from studies of patient-accessible medical records is that they can improve certain aspects of care, but they are unlikely to substantially improve health status. This probably reflects the inherent limitations of interventions that focus on information alone: a better-informed patient is not necessarily a healthier patient [27]. Future directions in patient-accessible electronic medical records will likely involve integrating educational strategies with behavioral strategies, so the medical record will be presented to patients in formats that are more comprehensible, more useful, and more likely to empower patients to care for themselves.

## Abbreviations

KCCQ: Kansas City Cardiomyopathy Questionnaire
MOS: Medical Outcomes Study
NYHA: New York Heart Association
SPPARO: System Providing Patients Access to Records Online

## References

[1] Ross Stephen E, Lin Chen-Tan (2003). The effects of promoting patient access to medical records: a review. J Am Med Inform Assoc.

[2] Ross A P (1986). The case against showing patients their records. Br Med J (Clin Res Ed).

[3] Stevens D P, Stagg R, Mackay I R (1977). What happens when hospitalized patients see their own records. Ann Intern Med.

[4] Hertz C G, Bernheim J W, Perloff T N (1976). Patient participation in the problem-oriented system: a health care plan. Med Care.

[5] Baldry M, Cheal C, Fisher B, Gillett M, Huet V (1986). Giving patients their own records in general practice: experience of patients and staff. Br Med J (Clin Res Ed).

[6] Golodetz A, Ruess J, Milhous R L (1976). The right to know: giving the patient his medical record. Arch Phys Med Rehabil.

[7] Cimino J J, Patel V L, Kushniruk A W (2001). What do patients do with access to their medical records?. Medinfo.

[8] Masys Daniel, Baker Dixie, Butros Amy, Cowles Kevin E (2002). Giving patients access to their medical records via the internet: the PCASSO experience. J Am Med Inform Assoc.

[9] Goldberg Harold I, Ralston James D, Hirsch Irl B, Hoath James I, Ahmed Kazi I (2003). Using an Internet comanagement module to improve the quality of chronic disease care. Jt Comm J Qual Saf.

[10] Green C P, Porter C B, Bresnahan D R, Spertus J A (2000). Development and evaluation of the Kansas City Cardiomyopathy Questionnaire: a new health status measure for heart failure. J Am Coll Cardiol.

[11] Brown J B, Boles M, Mullooly J P, Levinson W (1999). Effect of clinician communication skills training on patient satisfaction. A randomized, controlled trial. Ann Intern Med.

[12] Morisky D E, Green L W, Levine D M (1986). Concurrent and predictive validity of a self-reported measure of medication adherence. Med Care.

[13] Dimatteo M R, Sherbourne C D, Hays R D, Ordway L, Kravitz R L, Mcglynn E A, Kaplan S, Rogers W H (1993). Physicians' characteristics influence patients' adherence to medical treatment: results from the Medical Outcomes Study. Health Psychol.

[14] Kravitz R L, Hays R D, Sherbourne C D, Dimatteo M R, Rogers W H, Ordway L, Greenfield S (1993). Recall of recommendations and adherence to advice among patients with chronic medical conditions. Arch Intern Med.

[15] Hays R D, Kravitz R L, Mazel R M, Sherbourne C D, Dimatteo M R, Rogers W H, Greenfield S (1994). The impact of patient adherence on health outcomes for patients with chronic disease in the Medical Outcomes Study. J Behav Med.

[16] Verbeke Geert, Molenberghs Geert, Bickel P, Fienberg S, Krickenberg K, Olkin I (2000). Linear Mixed Models for Longitudinal Data.

[17] Bronson D L, O'meara K (1986). The impact of shared medical records on smoking awareness and behavior in ambulatory care. J Gen Intern Med.

[18] Bouchard RE, Tufo HM, Van Buren HC, Eddy WM, Twitchell JC, Bedard L (1973). The patient and his problem-oriented record. In Walker HK, Hurst JW, Moody MF (eds): Applying the problem-oriented system.

[19] Elbourne D, Richardson M, Chalmers I, Waterhouse I, Holt E (1987). The Newbury Maternity Care Study: a randomized controlled trial to assess a policy of women holding their own obstetric records. Br J Obstet Gynaecol.

[20] Homer C S, Davis G K, Everitt L S (1999). The introduction of a woman-held record into a hospital antenatal clinic: the bring your own records study. Aust N Z J Obstet Gynaecol.

[21] Glasgow Russell E, Boles Shawn M, Mckay H Garth, Feil Edward G, Barrera Manuel (2003). The D-Net diabetes self-management program: long-term implementation, outcomes, and generalization results. Prev Med.

[22] Katz Steven J, Moyer Cheryl A, Cox Douglas T, Stern David T (2003). Effect of a triage-based E-mail system on clinic resource use and patient and physician satisfaction in primary care: a randomized controlled trial. J Gen Intern Med.

[23] Glasgow Russell E, Bull Sheana S, Gillette Cynthia, Klesges Lisa M, Dzewaltowski David A (2002). Behavior change intervention research in healthcare settings: a review of recent reports with emphasis on external validity. Am J Prev Med.

[24] Stein E J, Furedy R L, Simonton M J, Neuffer C H (1979). Patient access to medical records on a psychiatric inpatient unit. Am J Psychiatry.

[25] Miller R D, Morrow B, Kaye M, Maier G J (1987). Patient access to medical records in a forensic center: a controlled study. Hosp Community Psychiatry.

[26] Bernadt M, Gunning L, Quenstedt M (1991). Patients' access to their own psychiatric records. BMJ.

[27] Noell J, Glasgow R E (1999). Interactive technology applications for behavioral counseling: issues and opportunities for health care settings. Am J Prev Med.

