# Supplementary material for: Providing a Web-based Online Medical Record with Electronic Communication Capabilities to Patients With Congestive Heart Failure: Randomized Trial
Source: J Med Internet Res. 2004 May 14;6(2):e12. doi: 10.2196/jmir.6.2.e12 (PMC1550594; doi:10.2196/jmir.6.2.e12)
Supplement: Supplementary file 1 [file jmir_v6i2e12_app1.ppt]

## Slide 1
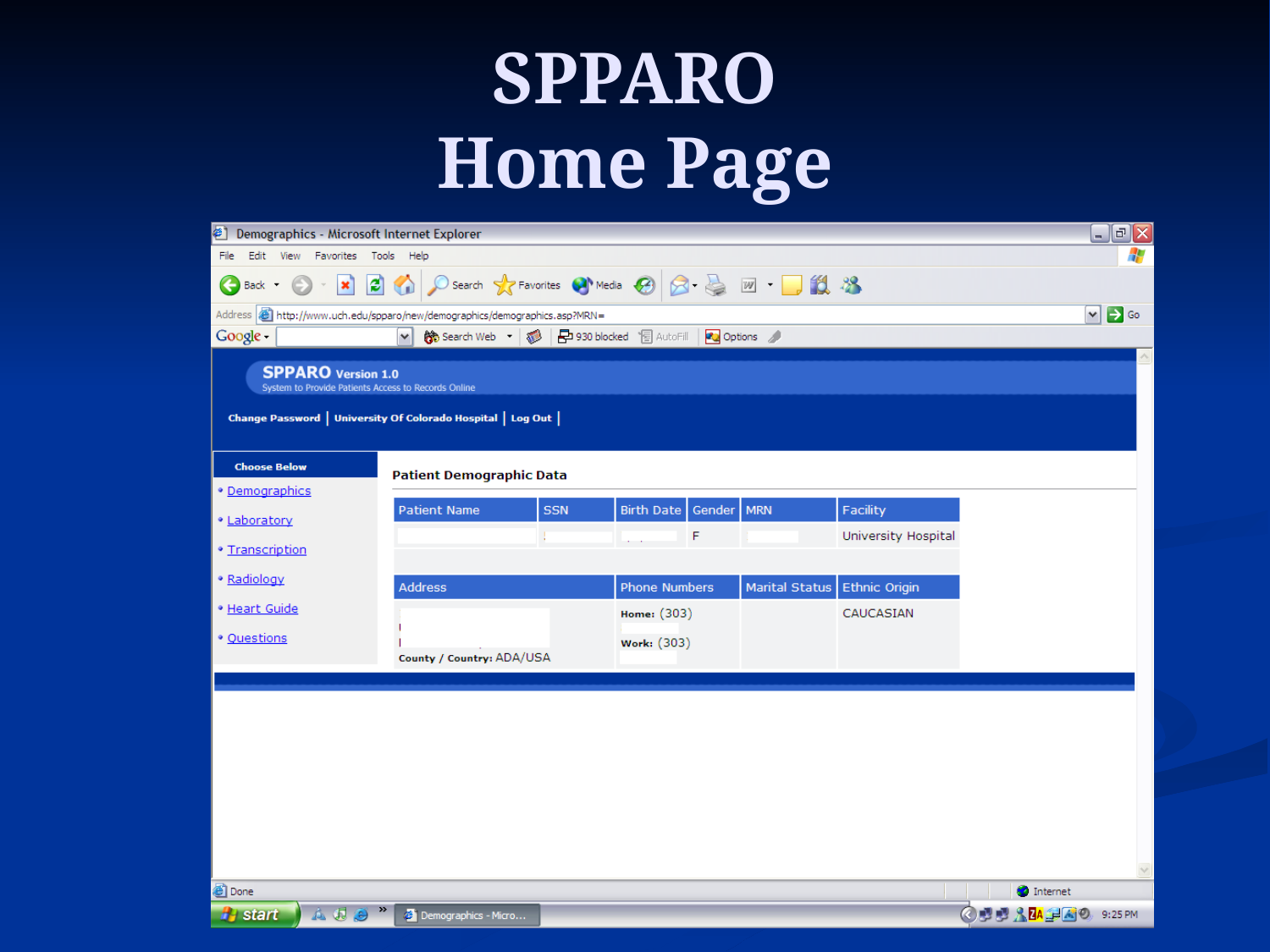

# SPPARO Home Page

## Slide 2
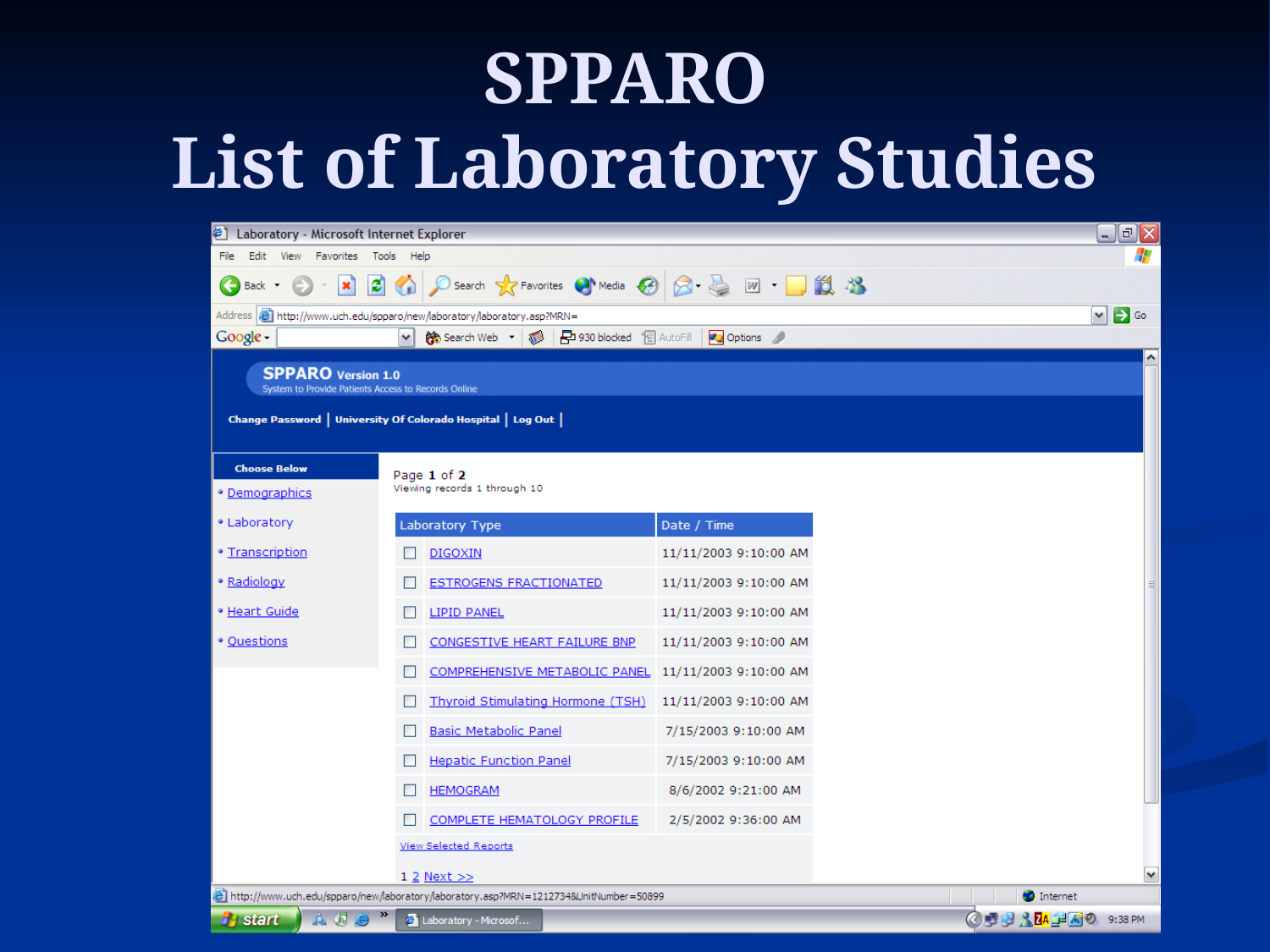

# SPPARO List of Laboratory Studies

## Slide 3
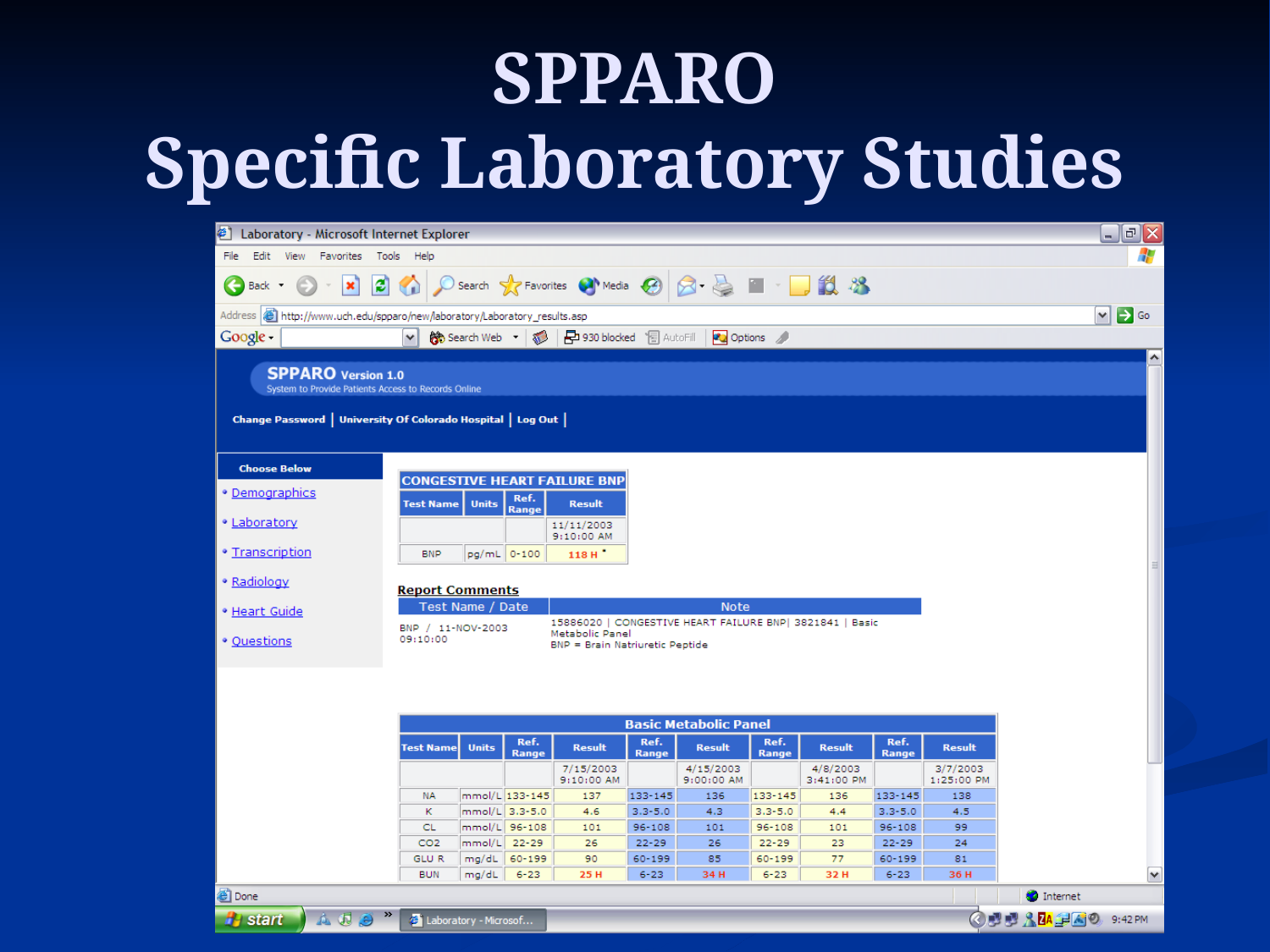

# SPPARO Specific Laboratory Studies

## Slide 4
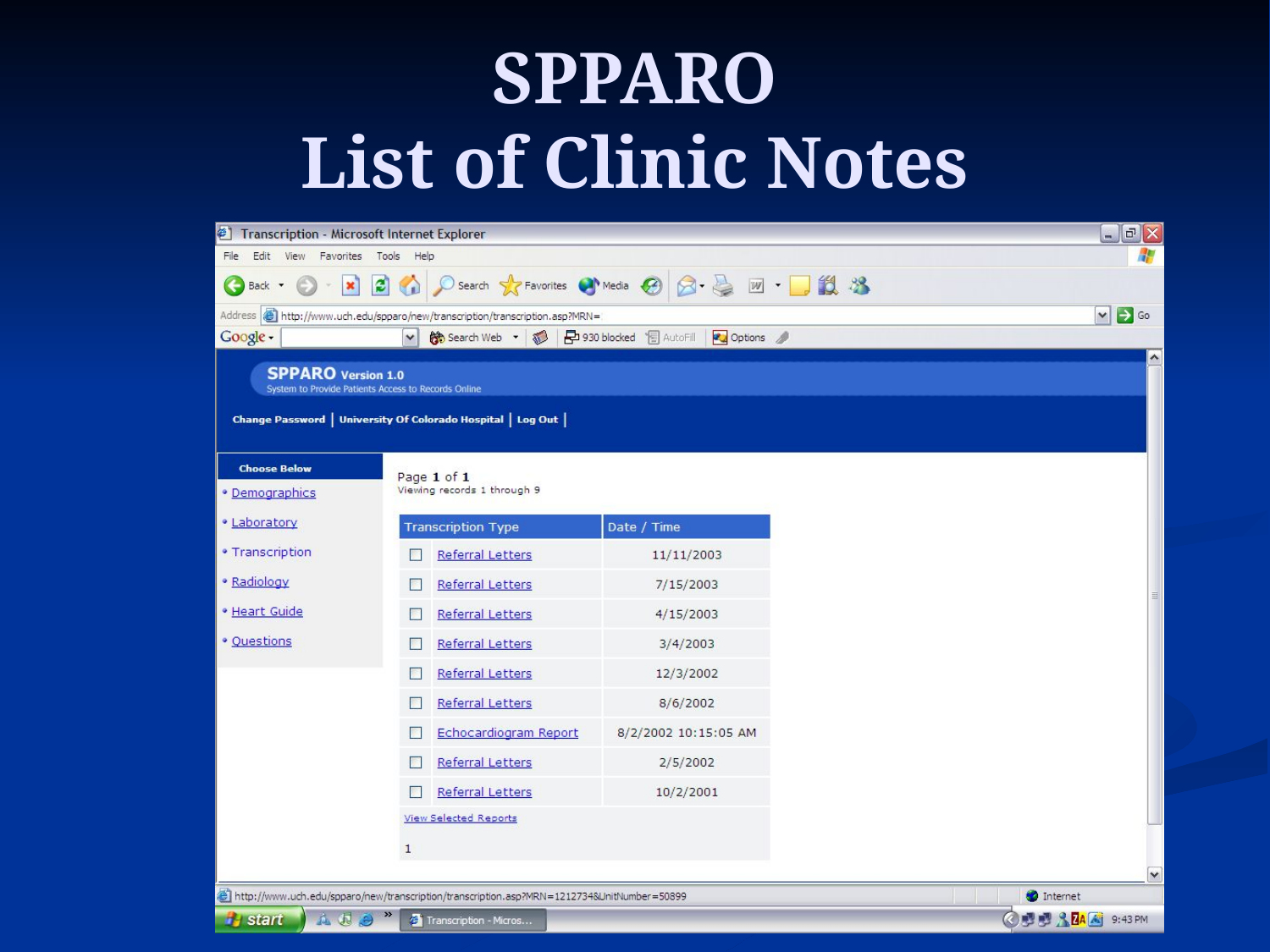

# SPPARO List of Clinic Notes

## Slide 5
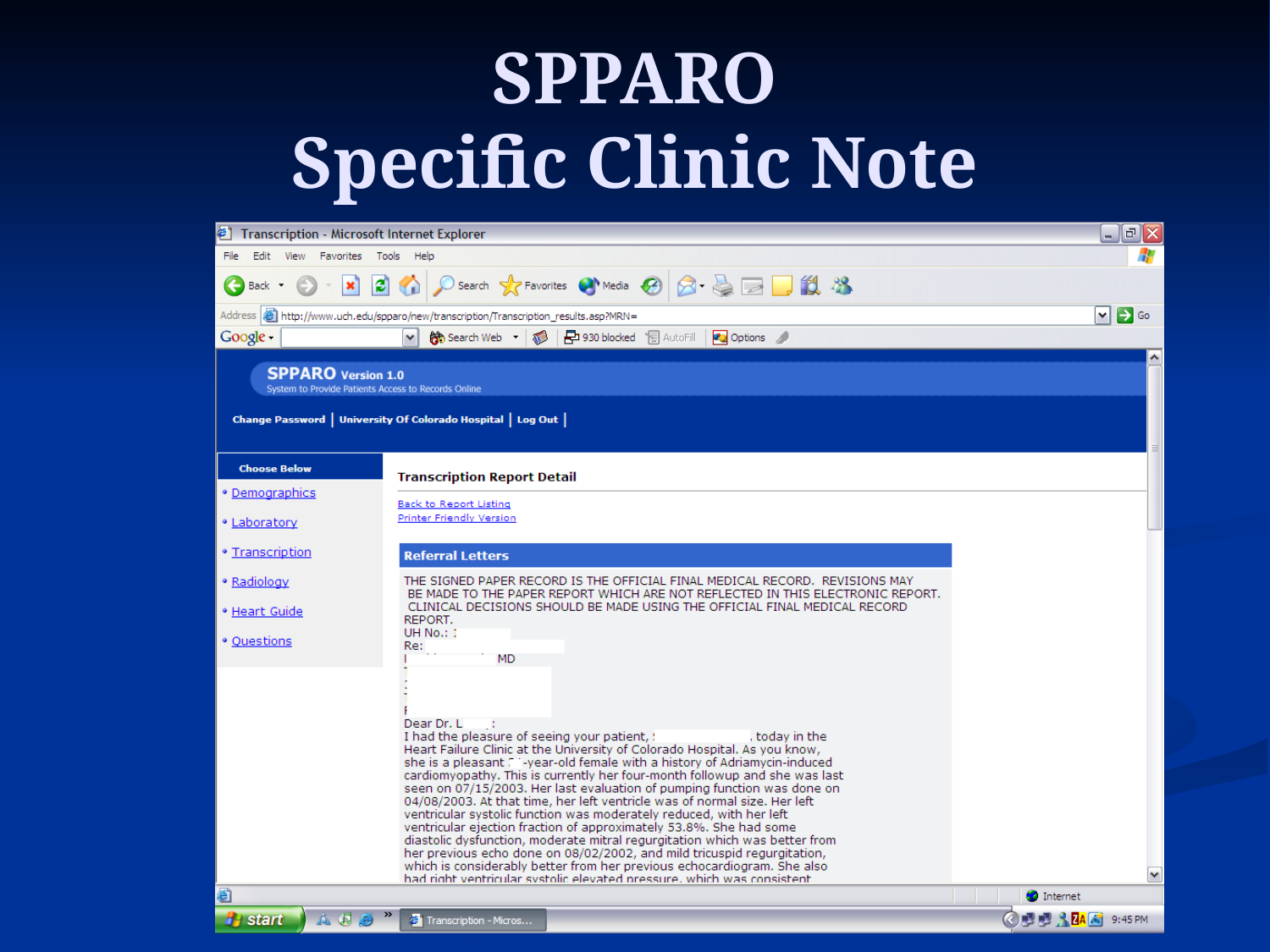

# SPPARO Specific Clinic Note

## Slide 6
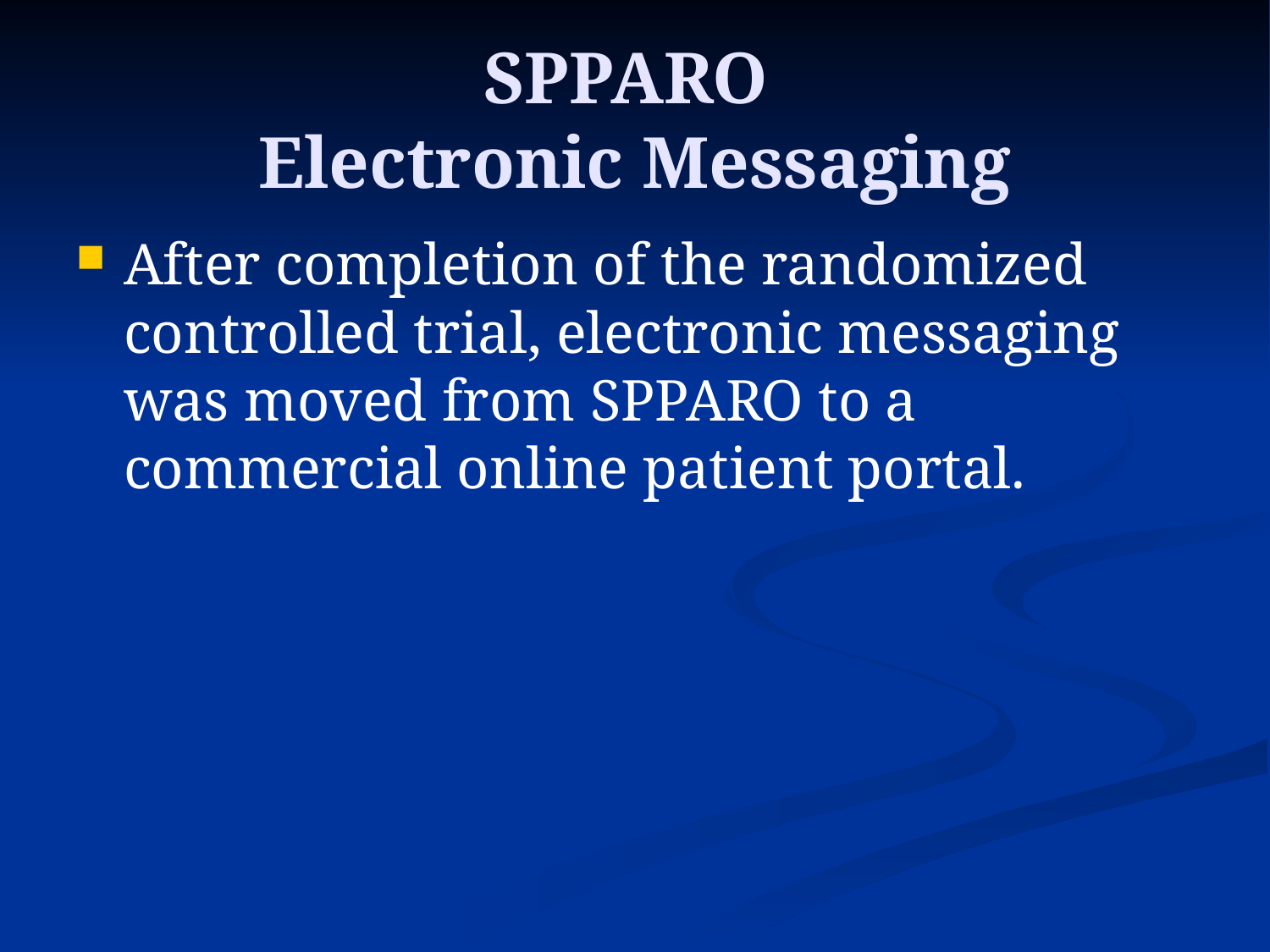

# SPPARO Electronic Messaging
After completion of the randomized controlled trial, electronic messaging was moved from SPPARO to a commercial online patient portal.
